# Species traits modify the species-area relationship in ground-beetle (Coleoptera: Carabidae) assemblages on islands in a boreal lake

**DOI:** 10.1371/journal.pone.0190174

**Published:** 2017-12-20

**Authors:** Aaron J. Bell, Iain D. Phillips, Scott E. Nielsen, John R. Spence

**Affiliations:** 1 Department of Renewable Resources, University of Alberta, Edmonton, Alberta, Canada; 2 Troutreach Saskatchewan, Saskatchewan Wildlife Federation, Moose Jaw, Saskatchewan, Canada; 3 Department of Biology, University of Saskatchewan, Saskatoon, Saskatchewan, Canada; 4 Water Quality Services, Integrated Water Services, Water Security Agency of Saskatchewan, Saskatoon, Saskatchewan, Canada; Inha University, REPUBLIC OF KOREA

## Abstract

Life-history traits influence colonization, persistence, and extinction of species on islands and are important aspects of theories predicting the geographical distribution and evolution of species. We used data collected from a large freshwater lake (1,413 km^2^) in central Canada to test the effects of island area and isolation on species richness and abundance of carabid beetles as a function of body size, wing length, and breeding season. A total of 10,018 individual beetles from 37 species were collected during the frost-free period of 2013 using transects of pitfall traps on 30 forested islands ranging in area from 0.2 to 980.7 ha. Life-history traits improved the predictive ability and significantly modified the shape of species-area and abundance-area curves. Abundance and richness of small-bodied (< 13.9 mm), macropterous (winged), and spring-breeding species decreased with island area and increased with isolation. In contrast, richness and abundance of larger-bodied (> 14.0 mm) and flightless species increased with area, but not isolation. Body size of female *Carabus taedatus* Fabricius, the largest-bodied species, was positively related to island area, while body size on the adjacent mainland was most similar to that on smaller islands. Overall, species with large body size and low dispersal ability, as indicated by flightlessness, were most sensitive to reductions in area. We suggest that large-bodied, flightless species are rare on small islands because habitat is less suitable for them and immigration rates are lower because they depend on freshwater drift for dispersal to islands.

## Introduction

The tendency for species number to increase with area (i.e., the species-area relationship, hereafter ‘SAR’) is one of the oldest and well-documented patterns in ecology [1,2]. The pattern holds true for a wide range of taxa and habitats [3] and thus has been referred to as one of ecology’s few ‘laws’ [4]. Numerous causal explanations have been offered to explain the SAR [3], including influence of a variety of deterministic (i.e., species traits) and stochastic (i.e., extinction and colonization) factors; however, there is little consensus about the relative importance of such factors [3]. Traditional niche theory, for example, emphasizes the joint importance of species traits (dispersal ability, niche-breadth, and fecundity) and habitat diversity in generating SARs [5]. In contrast, island biogeography theory ignores the functional importance of such traits and argues instead that SARs arise simply from dynamic colonization and extinction processes [6,7]. More recently, researchers have called for a more integrative approach that includes both stochastic and deterministic factors in modelling SARs [8,9].

One integrative approach to understanding factors underlying SARs is to include species traits as an additional parameter in the model (e.g., [10]). This approach invokes both concepts of niche and neutral theories of island biogeography by incorporating both deterministic and stochastic factors within a single model [10]. Furthermore, inclusion of species traits can improve the predictive power of SARs, while also identifying specific traits or combinations thereof that are important in conservation planning [10,11]. Comparing slopes for species with different traits, for example, can help to identify species that are most sensitive to changes in area that might occur through habitat loss or fragmentation [10,11]. While numerous studies have focused on how variation in traits between taxa may influence SARs [12–15], fewer have considered trait-specific variation at the species level [10,11,16]. Here, we consider for the first time variation in SARs on lake islands in relation to ecologically relevant traits of carabid beetles (Coleoptera: Carabidae).

Carabid beetles are well suited to studies of island ecology and have been widely employed in this context [17–21]. Carabid dispersal is influenced by wing length and wing muscle development [22–25], thus effects of differences in dispersal ability on patterns of island occupancy can be studied [22]. Likewise, both habitat use [24–26] and patterns of reproductive activity [27–29] vary among carabid species and may explain variation in carabid distributions on islands.

Variation in body size has often been studied for island faunas because it influences many characteristics associated with immigration potential, ecological interactions, and resource requirements [30]. Although variation in body size of vertebrates has been relatively well-studied on islands (with differential effects in relation to average body size termed the ‘island rule’, see [30–34]), there have been fewer studies of variation in body size among invertebrates (but see [35,36]), and we are unaware of any studies of intraspecific variation in carabid body size on true islands. In the context of habitat ‘islands’ in mainland systems, contiguous (i.e., larger) mature forests generally are characterized as having large-bodied species, whereas more disturbed and isolated (i.e., smaller) habitats are dominated by small-bodied species [37–40]. It is not well understood, however, whether differences in body size are related to habitat quality within these patches or patch area *per se*.

In this paper, we explored how patterns in carabid life-history traits are related to area and isolation of 30 islands in Lac la Ronge; a large (1,413 km^2^) freshwater lake in the boreal forest of northern Saskatchewan, Canada. We focused on beetle traits that are related to processes of colonization and extinction including body size, wing length, and breeding period. Species-area and abundance-area relationships were considered in relation to these traits and measurements of individual beetles were also analyzed to assess intraspecific variation in body size on the islands. Specifically, we tested the following predictions: 1) species traits modify the slope of species-area and abundance-area relationships; 2) species with large body size, flightlessness, and autumn-reproduction increase in abundance with island area; and 3) body size of carabid individuals increases intraspecifically with island area.

## Materials and methods

Permission to conduct this study was provided by the Saskatchewan Ministry of Environment.

### Site description

**Fig 1:**
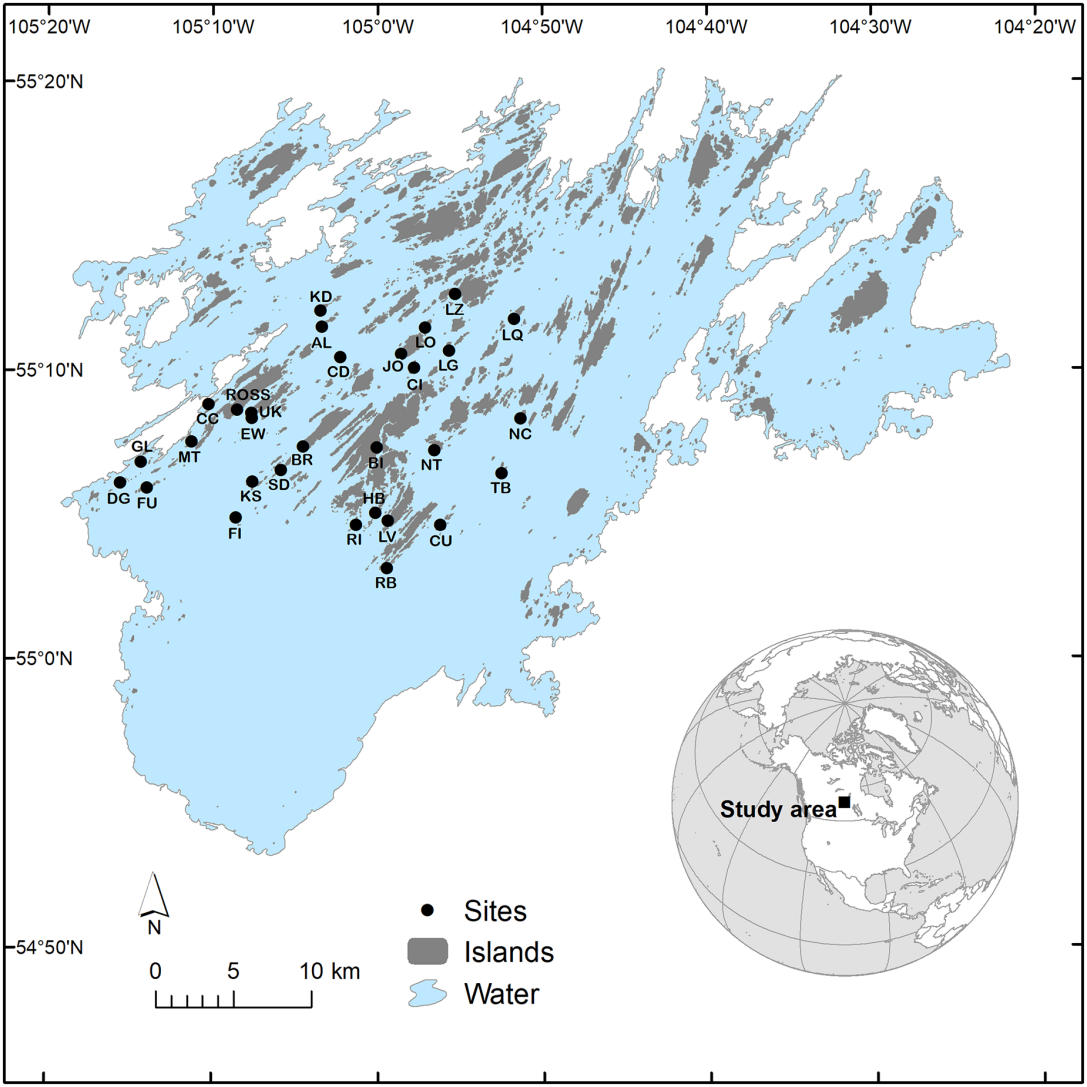
Map of the islands of Lac la Ronge, Saskatchewan. Black circles indicate the islands that were sampled. Refer to Table 1 for island abbreviations.

Lac la Ronge (55°06’ N, 105°01’ W) is a large (1,413 km^2^) lake located in the boreal forest of Saskatchewan, Canada (Fig 1). Approximately 10,200–9,000 years BP [41] this region was covered by Lake Agassiz, a huge glacial lake that formed at the margins of the retreating Laurentide Ice Sheet. The current remnant of that lake now called Lac la Ronge sits at the boundary of the Canadian Shield, and is thus surrounded by geologically distinct regions to the north and south. The southern reaches of the lake have few islands with sediments being mainly gravel, sand, and clay. In contrast, rugged igneous and metamorphic bedrock [42] is exposed in the northern and central reaches, giving rise to a collection of >1,300 islands. These islands are covered mainly by mixed and conifer-dominated forest, including some gaps surrounded by forest (see [43]), with sand beaches, bogs, and meadows comprising small areas on some islands. Except for a few small cabins, islands are not impacted by human activity.

Wildfire has been the primary post-glacial disturbance in the region. Parisien et al. [44] estimated that the fire return interval on the mainland was 99–104 years, but islands in the region have a much longer fire interval [45]. Although no work has specifically addressed the fire interval on the islands of Lac la Ronge, fire mapping in the area since 1980 [45] and the presence of late-successional species [46,47], such as *Picea glauca* (Moench) and *Abies balsamea* (Linnaeus) suggest that none of the study islands have burned recently.

### Sampling protocol

Carabids were sampled continuously between 2 June– 23 August, 2013 (the approximate frost-free period for La Ronge, Saskatchewan; http://climate.weather.gc.ca/climate_normals) on 30 islands, varying in area (0.2–980.7 ha) and distance to mainland (0.1–10.7 km; Table 1). We placed eight sleeved pitfall traps (11.2 cm diameter, see [48]) in similar habitats along 120 m transects on each island, for a total of 240 pitfall traps. Transect position and bearing was determined at random and small adjustments to the direction of the transect were made to ensure a minimum distance of 10 m from the shoreline. Traps were positioned along the transect with the first trap located at 7.5 m, and 15 m between subsequent traps to help ensure a more representative catch (see [49]). Each trap was filled with propylene glycol to a depth of 2–3 cm to kill and preserve captured beetles, and covered by a small plywood lid (15 x 15 cm) suspended from corner posts above the trap to exclude rainwater and debris. Transects were visited a total of five times at 14–17 day intervals to collect samples and replenish the preservative in each trap. Pitfall catches measure activity-density that is generally interpreted as a measure of relative abundance (hereafter called abundance; see [48]).

**Table 1:** Study islands on Lac la Ronge, total number of individuals (*n*), and the distribution of beetle traits observed on each island.

Island number	Island ID	Area (ha)	Distance to mainland (km)	*n*	small	large	winged	wingless	spring	autumn
1	EW	0.2	2.7	142	8	1	4	1	6	3
2	FI	0.3	6.0	589	14	0	9	0	8	6
3	HB	0.5	10.7	341	13	2	8	2	8	7
4	LG	0.6	8.9	365	12	3	8	3	10	5
5	AL	0.7	2.1	813	10	2	5	2	7	5
6	GL	0.7	0.4	670	11	2	7	2	7	6
7	CI	1.2	8.3	146	8	2	5	2	6	4
8	CU	1.5	7.6	452	12	0	7	0	8	4
9	RI	1.6	10.6	351	13	2	10	1	9	6
10	RB	2.5	7.2	407	12	2	9	2	9	5
11	FU	2.6	1.4	460	12	2	8	2	10	5
12	CD	3.2	3.9	163	8	2	5	2	5	5
13	KS	3.4	5.1	399	12	2	9	2	9	5
14	MT	7.5	0.4	267	8	2	4	2	5	5
15	SD	8.2	6.1	232	7	2	3	2	5	4
16	DG	10.3	0.5	406	7	2	4	2	5	4
17	LO	15.1	6.7	443	9	3	4	4	6	6
18	NC	19.3	7.4	43	5	3	2	3	5	3
19	TB	19.5	6.6	718	13	3	9	3	10	6
20	CC	21.1	0.1	74	6	2	3	2	4	4
21	LQ	26.9	7.2	110	8	2	5	2	5	5
22	KD	29.4	2.0	301	6	3	4	2	5	4
23	NT	43.2	10.3	85	12	2	7	3	8	6
24	UK	124.3	2.2	355	9	2	5	2	6	5
25	JO	130.2	6.6	396	7	3	4	3	6	4
26	LV	169.1	9.4	89	7	4	5	4	6	5
27	BR	255.1	5.8	559	10	3	6	4	7	6
28	LZ	289.2	6.2	268	7	3	4	3	6	4
29	ROSS	534.8	0.3	119	9	2	3	3	4	7
30	BI	980.7	8.1	255	10	3	4	4	5	8
*Sum*				10018						

List of islands and their respective area and distances to mainland. Distance to mainland (isolation), number of small- and large-bodied species, macropterous and flightless species, and spring and autumn-breeding species by island are also listed.

Because traits like body size and wing length are known to vary with the openness of habitat [38,40], forest canopy above each transect was measured using the line-intercept (0.1 m minimum increment) method for all woody species > 1 m height and diameter breast height (DBH) ≥ 4.5 cm. Proportion canopy cover was then calculated as the sum of canopy cover overlapping the transect divided by transect length. Because of forest gaps, canopy cover varied from 28 to 100% among transects; however, this variation was not related to island area (*r* = 0.20, *P* = 0.29).

### Life-history traits

Body length was measured as total length (tip of mandibles to apex of elytra) of specimens for each species and taken as a comparative estimate of body size. Preserved beetles were flattened, positioned dorsoventrally next to a ruler, and photographed using a Canon Rebel T3i digital camera and Tamron 90 mm macro lens. Beetle length was calculated using the ruler for calibration in each photo using ImageJ software [50]. To compare abundance and species richness of carabids by body size, species were categorized as ‘small-bodied’ (< 13.9 mm) or ‘large-bodied’ (> 14.0 mm) based on median body length for each species. The differences between large and small-bodied species corresponded to a break that divided the smallest and largest specimen most nearly in half (i.e., 3.5–24.5 mm). The following five common species that represent the range of sizes studied were selected to assess intraspecific variation in body size across islands: *Carabus taedatus* Fabricius (median: 21.6 mm), *Carabus chamissonis* (Fisher von Waldheim) (18.0 mm), *Pterostichus punctatissimus* (Randall) (16.6 mm), *Pterostichus adstrictus* (Eschscholtz) (12.1 mm), and *C*. *ingratus* (8.4 mm). A minimum of 20 males and 20 females of each species, chosen at random from each island, were measured, except in cases where fewer specimens in good condition were available. In an effort to better understand the island effect on body size, we also measured a sample for each of the above listed carabid species collected with pitfall traps from mainland sites near Lac la Ronge (see [43]).

Wing length of all specimens was determined by raising the elytra to check wing development, and species were classified with respect to wing length as either (1) macropterous, i.e., hind wings fully developed in all specimens; (2) all specimens flightless, either brachypterous or apterous; or (3) dimorphic, having both macropterous and flightless individuals (see [40]). Wing dimorphic species were excluded from analyses that required that flight ability was designated to a single category.

Carabids of temperate and boreal regions are generally classified as either spring-breeding or autumn-breeding species (hereafter called spring-breeders and autumn-breeders, [51]). Spring-breeders overwinter as adults and reproduce during early summer, while most autumn-breeders overwinter as larvae and complete development the following spring or summer with new adults breeding at earliest in late July. Although this oversimplifies some of the complexity in carabid life-cycles (see [52]), Zalewski [29] found that autumn-breeders were less common on small islands and suggested that they are more prone to extinction on islands because overwintering larvae are less tolerant to fluctuating environmental conditions than adults. Information about carabid breeding periods was obtained from the literature [51,53]. Because the life-cycle of *Cicindela longilabris* Say cannot be easily classified [53], it was omitted from the analysis of seasonal breeding in relation to island area.

### Data analysis

Interactions between life-history traits and island area in determining abundance and species richness were analyzed using negative binomial (generalized linear model) and linear regression, respectively. For species richness models, we included distance to mainland as a measure of isolation due to its general importance as a predictor of island diversity [6,7]. We also included canopy cover (one measure of habitat quality) as a covariate, and used a model selection procedure to determine whether the form of this covariate should be linear or quadratic. We used Akaike information criterion (AIC_c_) with small sample correction [54] (see S1 Table), choosing the candidate model with smallest AIC_c_ and thus the largest Akaike weight (*w*_*i*_) as the most supported. We then assessed the interaction between life-history traits and island area using results from that model. We included the number of trap days as a covariate to account for minor differences in trapping effort due to lost traps (see [43]). Residuals for models assessing species richness by island area met the assumptions of normality and equal variance.

All analyses were conducted in R statistical software [55]. Relationships between individual body size and island area were analyzed for all five species assessed using a linear mixed-effects model (*lme4* package) with island identity used as a random effect. Sex was included as a fixed effect in all models to account for larger body size of females. Statistical significance of the mixed model was evaluated using a likelihood ratio χ^2^ test comparing models that included island area with those that did not [56]. All comparisons of carabid body size relationships between islands and the mainland were based on 95 confidence intervals (CI) with non-overlapping confidence intervals considered to indicate populations of significantly different body size. Confidence intervals were constructed using non-parametric bootstrapping with replacement in which body size for each species was randomly sampled 10,000 times from the original data pool.

## Results

### Effects of species traits

**Fig 2:**
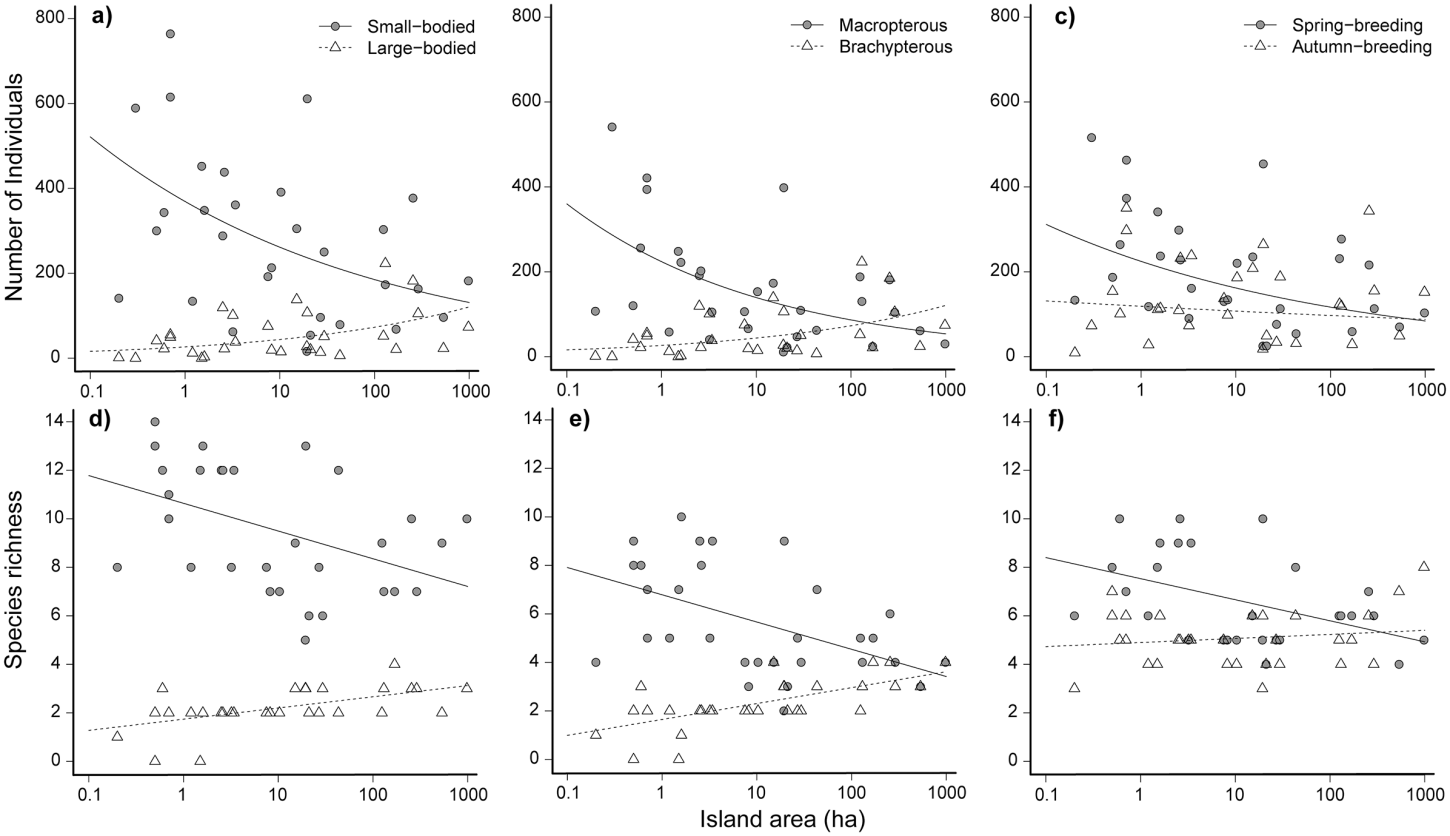
Regression plots of species abundance (a-c) and richness (d-f) by island area (log_10_) for species traits: Body size, wing length, and breeding season. Plots include canopy cover and distance to mainland set at the mean.

In total, 10,018 adult carabids representing 37 species were collected on the 30 islands studied in Lac la Ronge (Table 1). Neither species richness, nor abundance varied with island area (*P* = 0.47, R^2^ = 0.02, and *P* = 0.25, D^2^ = 0.08, respectively). However, when species traits and their interactions with island area were incorporated in the model, variance explained increased to 26–52% and 40–83% for a range of abundance and species richness models, respectively (see Table 2). Furthermore, a significant interaction between species traits and island area revealed that abundance and richness of small-bodied and macropterous species increased with island area, while the opposite pattern was observed for large-bodied and flightless species (see Fig 2). A list of carabid species and their associated life-history traits is given in S1 Table.

**Table 2:** Most supported regression models describing abundance (negative binomial) and species richness (linear) on the islands of Lac la Ronge.

Model		Model Structure	*K*	*P*	R^2^ adj.	D^2^
a.	Body size					
	1 abundance	canopy+area*body size+trap days	6	<0.0001		0.52
	2 species richness	canopy+area*body size+distance to mainland+trap days	7	<0.0001	0.83	
b.	Wing length					
	1 abundance	canopy+area*wing length+trap days	6	<0.0001		0.42
	2 species richness	canopy+canopy^2+area*wing length+distance to mainland+trap days	8	<0.0001	0.66	
c.	Breeding season					
	1 abundance	canopy+canopy^2+area*breeding season+trap days	7	0.0010		0.26
	2 species richness	canopy+canopy^2+area*breeding season+distance to mainland+trap days	8	<0.0001	0.40	

Model name, structure, total parameters (K), significance level (*P*), adjusted R^2^, and % deviance explained (D^2^) are provided.

#### Carabid abundance

A linear model of canopy structure provided the most supported explanation of abundance for body size and wing length models, while the non-linear (quadratic) canopy structure model was the most supported for the breeding season model (see S2 Table). However, after controlling for the effects of canopy cover, we found clear support for relationships between both body size and wing length and island area in local abundance of species (S3 Table). Models that included body size and wing length were also significant (*P* < 0.001, D^2^ = 0.52 and *P* < 0.001, D^2^ = 0.42, respectively, Table 2) with interactions between body size and island area and wing length and island area both supported (*P* < 0.001). Abundance of large-bodied, flightless species increased with island area, while abundance of small-bodied, macropterous species was inversely related to island area (see Fig 2a and 2b).

The model including breeding season was also significant (*P* = 0.001 and D^2^ = 0.26, Table 2, Fig 2c), although the interaction between breeding season and island area was not significant (*P* = 0.15). Abundance of spring-breeders was inversely related to island area (*P* = 0.004), while abundance of autumn-breeders did not vary with island area (*P* = 0.49).

#### Species richness

A linear model of canopy cover was the most supported explanation of richness for the body size model, while a non-linear (quadratic) term was most supported for both the wing length and breeding season models of richness (S2 Table). Models including body size and wing length were significant (*P* ≤ 0.001, R^2^ = 0.83, and *P* ≤ 0.001, R^2^ = 0.66, Table 2), with an interaction supported between body size and island area and wing length and island area (*P* ≤ 0.001, Fig 2d and 2e). Richness of large-bodied and flightless species increased with island area, whereas the opposite pattern was observed for small-bodied and macropterous species (see Fig 2d and 2e). Similarly, the breeding season model and an interaction between breeding season and island area were significant in explaining richness (*P* ≤ 0.001, R^2^ = 0.38, Table 2, see Fig 2f). Richness of spring-breeders was inversely related to area (*P* = 0.007), although richness of autumn-breeding species did not vary with island area (*P* = 0.360, Fig 2f).

Distance to mainland was significant in each of the models relating species richness to island area and species traits (see S3 Table). However, contrary to predictions of island biogeography theory, richness of small-bodied (*P* = 0.046), macropterous (*P* = 0.021), and spring breeding (*P* = 0.019) species increased with distance to mainland. This pattern was not observed for large-bodied, flightless, and autumn-breeding species for which richness did not vary with distance to mainland (S3 Table).

### Intraspecific variation in body size

Body size was measured for a total of 3,204 individuals from five species. The two smallest species, *P*. *adstrictus* and *C*. *ingratus*, were sufficiently abundant on nearly all 30 islands to provide sufficient samples for the full range of island sizes. In contrast, the three larger species, *P*. *punctatissimus*, *C*. *chamissonis*, and *C*. *taedatus*, were absent from two, six, and 19 of the islands, respectively, and tended to be absent or lower in abundance on smaller islands. For example, aside from a single individual collected on island ‘LG’ (Table 1), *C*. *taedatus* was absent from samples from the 14 smallest islands (≤ 7.5 ha).

**Fig 3:**
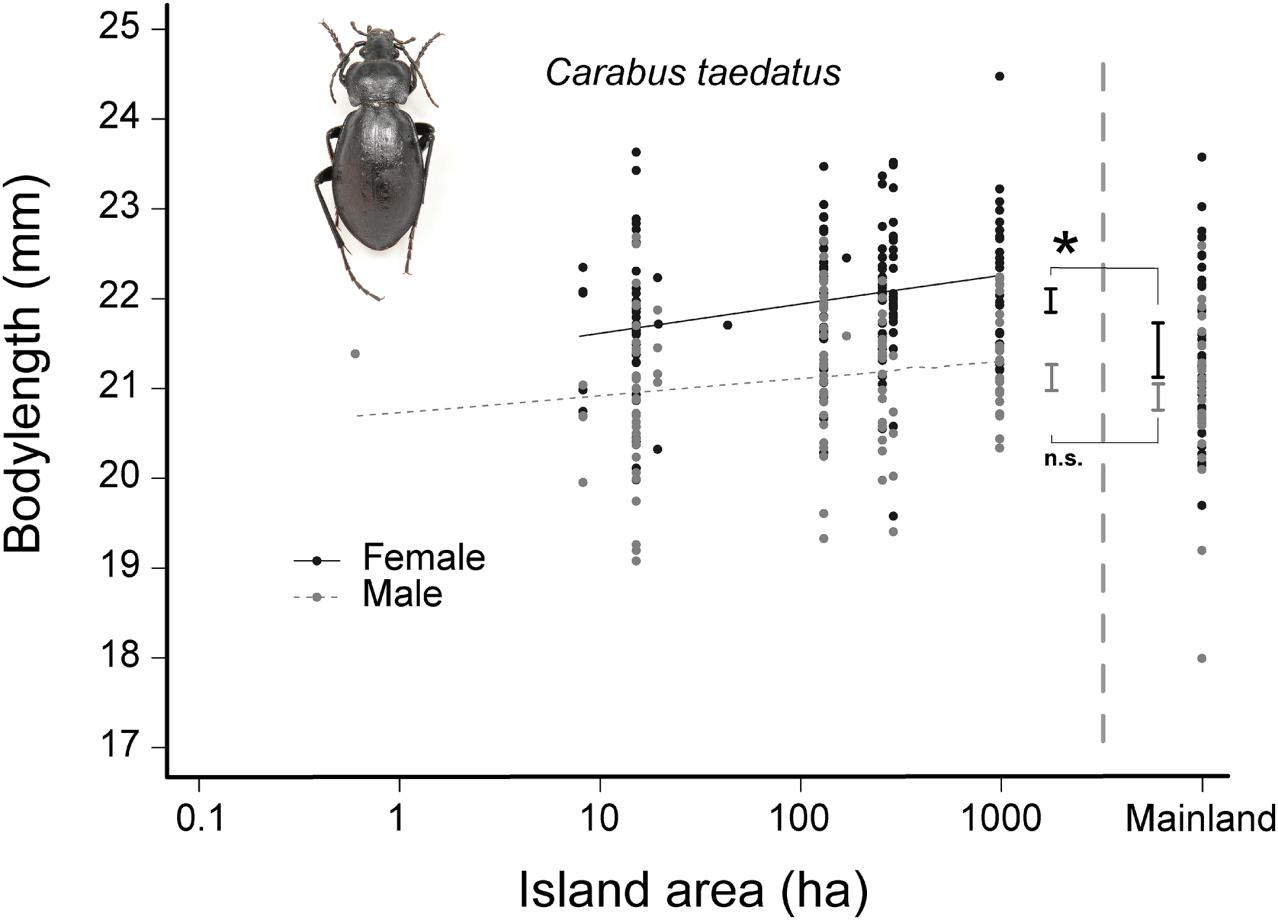
Regression plot of intraspecific body size and island area (log_10_) for *Carabus taedatus*. Body size measured from a sample of carabids on the mainland is shown on the right side of the graph. The slope of the regression line differs significantly different from zero for females (*P* = 0.002, χ^2^ = 9.50, df = 1) but not for males (*P* = 0.12, χ^2^ = 2.37, df = 1). Body size for females was larger on the islands (CI [21.85–22.11 mm], *n* = 153) than on the corresponding mainland (CI [21.13–21.73 mm], *n* = 36) but not for males.

As is typical for carabids, females were significantly larger than males for each species studied (Table 3). Body size was similar between islands and the mainland for four of the five species (see S1 Fig). However, body size varied significantly with island area for the largest species, *C*. *taedatus* (*P* = 0.003, χ^2^ = 8.89, df = 2). However, this pattern was due entirely to variation in size of females (Fig 3; *P* = 0.002, χ^2^ = 9.50, df = 1), which also showed greater range in body size ($\bar{\text{x}}$ = 22.0 ± 0.06 SE mm, 19.58–24.48 mm, *n* = 153) compared to males ($\bar{\text{x}}$ = 21.1 ± 0.07 SE mm, 19.08–22.69 mm, *n* = 122). Finally, female body size in *C*. *taedatus* was significantly larger on the islands (CI [21.85–22.11 mm], *n* = 153) than in samples from the mainland (CI [21.13–21.73 mm], *n* = 36), although as above, males sizes were similar.

**Table 3:** Estimated coefficients (β), standard errors (SE), and significance level of likelihood ratio tests for linear mixed-models assessing the effect of (log_10_) island area on intraspecific body size of five carabids species.

species	intercept		area		sex (male)		likelihood ratio χ^2^ test
	β	SE	β	SE	β	SE	
*C*. *taedatus*	21.4	0.158	0.255	0.069	-0.835	0.092	0.003
*C*. *chamissonis*	18.4	0.087	0.029	0.052	-1.08	0.062	0.570
*P*. *punctatissimus*	16.7	0.089	0.007	0.051	-0.121	0.077	0.880
*P*. *adstrictus*	12.2	0.046	-0.011	0.031	-0.235	0.045	0.720
*C*. *ingratus*	8.66	0.043	-0.016	0.027	-0.331	0.038	0.570

## Discussion

Neutral theories (e.g., [7,57]) challenge the traditional understanding that life-history traits are causally related to spatial distributions of species. Recent attempts to include both stochastic and deterministic factors in distribution models have, however, shown promise in providing better predictions of distributions [10,58]. Our results, along with those from several other recent studies [10,11,16], clearly demonstrate that species traits can be useful additions in species-area and abundance-area modelling. Predictive power of both our species-area and abundance-area models for ground-beetles was significantly improved with inclusion of species traits (Table 2). Furthermore, the strong interaction between species traits and island area revealed opposing patterns for two of the three traits examined: species richness and abundance of small-bodied, macropterous species was negatively related to island area, while the opposite pattern was observed for large-bodied, flightless species. In fact, we did not detect a positive SAR if species traits were not considered, likely because of the associated divergent response in richness and abundance (see [43]). Thus, inclusion of species traits in SAR modelling can help reveal complex interactions between species traits and area, as well as reconcile both niche theory and neutral theory in a single framework [10].

Large-bodied carabids were missing from many small islands and generally less abundant when present, with the opposite true for smaller-bodied species. Similar findings have been reported in relation to size of forest patches [10,59]. Species richness and abundance of large-bodied carabids is also known to vary with successional stage [38] and level of disturbance [37,59–64], with large-bodied species being more abundant in older forests and less disturbed, closed-canopy habitats. Interestingly, we found a residual relationship between body size and island area after controlling for habitat variation associated with canopy cover within mature island forests. Together, these findings suggest that both habitat quality and patch size are important for persistence of large-bodied forest specialists.

Local extinction appears to be a generally important process structuring species composition of island communities [7,65]. Carabid populations are generally short-lived with local extinctions occurring on decadal time scales [66–68]. Thus, it is possible that even small differences in extinction risk related to body size could contribute to the body size and island area associations observed in our study. Below, we suggest three lines of evidence supporting the inference that large-bodied carabids are more prone to extinction on small islands.

First, large-bodied species tend to have smaller populations, even among arthropods [69] and are thus more prone to local extirpation on small islands [7]. In our study, each of the three large-bodied species (*C*. *taedatus*, *C*. *chamissonis*, *P*. *punctatissimus*) collected in sufficient numbers were absent in samples from some small islands, although smaller-bodied species were more widely distributed across the island area gradient. The largest of these species, *C*. *taedatus*, was represented by a single individual among the 14 smallest islands (≤ 7.5 ha), but was collected commonly on ten of the 16 largest islands, suggesting that smalls islands rarely sustain populations of this species. Similar patterns were reported in a large-scale study of collembolans in Europe, in which the four largest-bodied species were absent from the islands, but occurred on the nearby mainland [36].

Second, large-bodied species, such as those mentioned in the previous paragraph, are typically flightless in the boreal forest and thus may be ‘rescued’ more infrequently from local extinction on islands by dispersal events. Immigration of carabids over water indicates that macropters dominate, even in drift material [70,71], suggesting that most drifting carabids are blown into the water from flight, and thus drift is less probable for large, flightless species. Our general observations of fewer flightless species on the islands are supported by other studies of carabids in freshwater systems [21,72] (but see [19,71]) and may result from occasional local extirpation on small islands with more infrequent re-colonization.

Third, we observed that body size in females of *C*. *taedatus*, the largest species examined, varied directly with island area. Studies of intraspecific variation in body size of carabids are limited, although two published examples suggest that beetles from less suitable habitats are smaller: 1) *Carabus nemoralis* declined significantly in body size towards the city center of an urban-rural gradient [62] and 2) four large-bodied species (ca. 21–30 mm) declined in body size in response to forest thinning and disturbance of ground vegetation in forests, although this effect was not observed among two smaller-bodied species (11.5–14.5 mm, [73]).

In our study, the relationship between island area and body size in *C*. *taedatus* was strongest in females, possibly due to sex-specific resource requirements associated with breeding. A general relationship between diet deficiency and body size is well-established in insects [74,75] and in carabids food composition influences egg number and size [76]. In general, female carabids invest in reproduction only after basic energy demands have been met, and because full reproductive potential is rarely achieved, Lövei & Sunderland [77] concluded that food shortages are common. Thus, the smaller body size observed for *C*. *taedatus* females on small islands likely reflects more limited food availability than on larger islands. Of course, this hypothesis assumes that these populations are long-lived enough for such selection to act.

Reduced body size on small islands has also been reported for the tenebrionid beetle *Asida planipennis* in the western Mediterranean [35] and for collembolans in island-mainland systems of southern Europe [36]. These studies, together with ours, broaden application of the ‘island rule’ to include some invertebrates. We observed that body size of *C*. *taedatus* was largest on the largest islands and was, in turn, reduced in size on the smallest islands and adjacent mainland. These findings are consistent with Palmer [35] who observed the largest body size of *A*. *planipennis* on islands 11.5 km^2^ in area with a reduction in body size for islands beyond or below this size (see also [30] for discussion on optimal body size). Future studies will help determine if there are patterns of size variation for invertebrates on islands and what factors may be involved in maintaining them.

It is important to note that while we considered species traits separately here, they are undoubtedly interrelated. Indeed, much of the focus on body size in ecological studies of islands relates to the influence of body size over other important characteristics of island faunas, such as immigration potential, ecological interactions, and resource requirements [30]. Similarly, a major challenge in studying dispersal relates to the multiple covarying traits (body size, sex, condition, and behaviour) that influence its propensity (see [78–80]). It is therefore likely that the effects of body size and wing length act in concert to shape carabid distributions in our study.

Contrary to predictions of the theory of island biogeography [6,7], we observed a positive relationship between richness and distance to mainland (isolation) for small-bodied, macropterous, and spring-breeding carabids. Although these findings seemed initially surprising, similar increases in species richness with isolation have also been reported for invertebrates in other island systems [19,81]. We offer two possible explanations for these patterns. First, greater flight potential of macropterous species (which in our study also tend to be small-bodied, spring breeders) allows these species to colonize more distant islands than their flightless counterparts. Second, isolated islands may experience less top-down control by vertebrate predators [81]. For example, Jonsson et al. [81] suggested that predation by birds occurs less frequently on isolated islands because they are suboptimal foraging sites compared to nearby habitats [82]. Although we have no direct evidence of predation on invertebrates, by-catch of small rodents (*Peromyscus maniculatus* and *Sorex* spp.) in our pitfall samples indicates that abundance of these insectivores decrease with distance to mainland (n = 99, r = − 0.36, *P* = 0.05). Predation by small mammals can significantly alter island invertebrate assemblages [83], thus, it is possible that the increase in richness with distance to mainland is related to fewer insectivorous predators on the most distant islands.

Our observation of negative abundance-area and species-area relationships for small-bodied species is also at odds with predictions of the theory of island biogeography. In a related study [43], we showed that the majority of negative co-occurrences between carabids on these islands involved large and small-bodied carabids. It is generally though that competition does not significantly influence carabid assemblages except through intra-guild predation [84] or at high densities [85] as may be expected on true islands. However, the density compensation hypothesis [86] posits that higher densities on small islands may arise when there are fewer large-bodied species, predators, competitors, or more stable environments relative to large islands. It is therefore possible that higher abundance and richness of small-bodied species on the smallest islands occurs because regulatory control over small-bodied species by their large-bodied counterparts is relaxed on these islands.

Zalewski [29] hypothesized that autumn-breeding carabid species that overwinter as larvae are more vulnerable to environmental fluctuations than are spring-breeders. Although we observed an effect of breeding season, this pattern was driven entirely by higher abundance and richness of spring-breeding species on the smallest islands. In contrast, abundance and richness of autumn-breeding species was not influenced by island area, prompting us to reject Zalewski’s hypothesis that autumn-breeding species are more sensitive to less stable environments on small islands.

## Conclusions

Our study, together with others, suggests that combining aspects of both niche theory and neutral theory provides better explanations for biological distributions than does either theory alone (Kadman and Allouche 2007; Lomolino and Brown 2009; Öckinger et al. 2010; Franzén et al. 2012; Dondina et al. 2016). Large-bodied, flightless carabids were less frequently captured on small islands indicating lower abundance. Although body size did not vary with island area for most species, females of *C*. *taedatus*, the largest-bodied species in our study, were larger on the largest islands. Together, these findings suggest that species with traits like large body size and flightlessness are sensitive to reductions in island area. In contrast, abundance and richness of small-bodied, macropterous, and spring-breeding carabids were inversely related to island area, possibly due to reductions in vertebrate predation and intra-guild predation by large-bodied carabid species on the smallest islands. Overall, our study supports the notion that life-history traits influence the distribution of carabid beetles on lake islands, and that such understanding can be used in accordance with classic SAR modelling to better understand island distributions.

## Supporting information

S1 Dataset Carabid body size data. (CSV)

S1 Fig Regression plots of intraspecific body size and island size (log_10_) for (A) *Calathus ingratus*, (B) *Pterostichus adstrictus*, (C) *Pterostichus punctatissimus*, and (D) *Carabus chamissonis*. Females (black dots, solid line) and males (grey dots, dotted line) are shown separately. Subsamples from the mainland are shown on the right side of the graph.
(TIF)

S1 Table List of candidate regression models. (DOCX)

S2 Table List of carabid species collected on the islands of Lac la Ronge. (DOCX)

S3 Table Estimated coefficients for linear regression models comparing abundance and proportion of species by life-history trait. (DOCX)

S1 Text Carabid identification and voucher locations. (DOCX)
