# Supplementary material for: Species traits modify the species-area relationship in ground-beetle (Coleoptera: Carabidae) assemblages on islands in a boreal lake
Source: PLoS One. 2017 Dec 20;12(12):e0190174. doi: 10.1371/journal.pone.0190174 (PMC5738139; doi:10.1371/journal.pone.0190174)
Supplement: S1 Table — (DOCX) [file pone.0190174.s003.docx]

**S1 Table.** List of candidate regression models, number of parameters (*K*), AIC_c_ scores, differences among AIC_c_ scores to the top model (Δ*_i_*), and AIC_c_ weights (*w_i_*) used to control for the effects of canopy in body size, wing-length, and breeding season for analyses of life-history traits of carabids on the islands of Lac la Ronge.

| Model #/model name | | | *K* | AIC_c_ | Δ*_i_* | *w_i_* | Model #/model name | | | *K* | AIC_c_ | Δ*_i_* | *w_i_* |
| --- | --- | --- | --- | --- | --- | --- | --- | --- | --- | --- | --- | --- | --- |
| *Species richness* | | | | | | | *Abundance* | | | | | | |
| a. | Body size model | |  |  |  |  | d. | Body size model | |  |  |  |  |
|  | 1 | Linear canopy | 7 | 244.1 | 0 | 0.58 |  | 1 | Linear canopy | 6 | 695.9 | 0 | 0.53 |
|  | 2 | Non-linear canopy | 8 | 244.7 | 0.7 | 0.42 |  | 2 | Non-linear canopy | 7 | 696.2 | 0.3 | 0.47 |
| b. | Wing-length model | |  |  |  |  | e. | Wing-length model | |  |  |  |  |
|  | 1 | Linear canopy | 7 | 225.8 | 1.7 | 0.30 |  | 1 | Linear canopy | 6 | 658.6 | 0 | 0.60 |
|  | 2 | Non-linear canopy | 8 | 224.1 | 0 | 0.70 |  | 2 | Non-linear canopy | 7 | 659.4 | 0.8 | 0.40 |
| c. | Breeding season model | |  |  |  |  | f. | Breeding season model | |  |  |  |  |
|  | 1 | Linear canopy | 7 | 218.7 | 0.4 | 0.45 |  | 1 | Linear canopy | 6 | 724.5 | 1.1 | 0.37 |
|  | 2 | Non-linear canopy | 8 | 218.3 | 0 | 0.55 |  | 2 | Non-linear canopy | 7 | 723.4 | 0 | 0.63 |
