# Supplementary material for: Species traits modify the species-area relationship in ground-beetle (Coleoptera: Carabidae) assemblages on islands in a boreal lake
Source: PLoS One. 2017 Dec 20;12(12):e0190174. doi: 10.1371/journal.pone.0190174 (PMC5738139; doi:10.1371/journal.pone.0190174)
Supplement: S2 Table — (DOCX) [file pone.0190174.s004.docx]

**S2 Table.** List of carabid species collected on the islands of Lac la Ronge, their wing length (m-macropterous, b-brachypterous, d-dimorphic), median body size, breeding period, and the numbers caught on the 15 smallest islands (0.2 – 8.2 ha) and 15 largest islands (10.3 – 980.7 ha).

| Species List | Wing Length | Body Size | Breeding Period | Small Islands | Large Islands |
| --- | --- | --- | --- | --- | --- |
| *Agonum affine* Kirby | m | 8.6 | spring | 1 | 1 |
| *A. corvus* (LeConte) | m | 8.8 | spring | 0 | 1 |
| *A. gratiosum* (Mannerheim) | m | 7.8 | spring | 28 | 5 |
| *A. melanarium* Dejean | m | 9.0 | spring | 14 | 0 |
| *A. propinquum* (Gemminger & Harold) | m | 7.2 | spring | 0 | 1 |
| *A. retractum* (LeConte) | d | 7.1 | spring | 669 | 250 |
| *A. sordens* (Kirby) | m | 5.9 | spring | 9 | 9 |
| *Amara erratica (*Duftschmid) | m | 7.6 | spring | 1 | 0 |
| *A. littoralis* Mannerheim | m | 8.0 | spring | 1 | 0 |
| *A. patruelis* Dejean | m | 8.7 | spring | 3 | 0 |
| *Bembidion bimaculatum* (Kirby) | m | 6.4 | autumn | 0 | 1 |
| *Bradycellus lugubris* (LeConte) | m | 6.7 | spring | 2 | 0 |
| *Calathus ingratus* Dejean | d | 8.4 | autumn | 1164 | 922 |
| *Carabus chamissonis* (Fisher von Waldheim) | b | 18.0 | spring | 291 | 400 |
| *C. taedatus* Fabricius | b | 21.6 | spring | 10 | 285 |
| *Calosoma frigidum* Kirby | m | 22.2 | spring | 1 | 1 |
| *Cicindela longilabris* Say | m | 16.0 | 3 year cycle | 0 | 1 |
| *Cymindis cribricollis* Dejean | d | 9.7 | autumn | 12 | 13 |
| *C. unicolor* Kirby | b | 8.8 | autumn | 0 | 6 |
| *Elaphrus clairvillei* Kirby | m | 9.1 | spring | 1 | 1 |
| *Harpalus fulvilabris* Mannerheim | d | 10.0 | autumn | 12 | 2 |
| *H. laevipes* Zetterstedt | m | 11.3 | autumn | 1 | 0 |
| *Loricera pilicornis* (Fabricius) | m | 7.8 | spring | 4 | 1 |
| *Miscodera arctica* Mannerheim | m | 8.0 | autumn | 5 | 3 |
| *Patrobus foveocollis* (Eschscholtz) | d | 9.8 | autumn | 0 | 4 |
| *P. septentrionis* Dejean | m | 10.0 | autumn | 2 | 0 |
| *Platynus decentis* (Say) | m | 11.3 | spring | 644 | 362 |
| *P. mannerheimii* (Dejean) | m | 12.1 | spring | 0 | 1 |
| *Pterostichus adstrictus* (Eschscholtz) | m | 12.1 | spring | 1796 | 858 |
| *P. brevicornis* (Kirby) | b | 5.5 | autumn | 0 | 2 |
| *P. pensylvanicus* (LeConte) | m | 12.1 | spring | 125 | 76 |
| *P. punctatissimus* (Randall) | b | 16.6 | autumn | 255 | 369 |
| *Stereocerus haematopus* (Dejean) | m | 11.7 | autumn | 378 | 352 |
| *Syntomus americanus* (Dejean) | d | 3.5 | spring | 12 | 0 |
| *Synuchus impunctatus* (Say) | d | 9.7 | autumn | 294 | 272 |
| *Trachypachus holmbergi* Mannerheim | m | 4.8 | spring | 61 | 19 |
| *Trechus apicalis* Motschulsky | d | 4.3 | autumn | 1 | 3 |
