# Supplementary material for: Species traits modify the species-area relationship in ground-beetle (Coleoptera: Carabidae) assemblages on islands in a boreal lake
Source: PLoS One. 2017 Dec 20;12(12):e0190174. doi: 10.1371/journal.pone.0190174 (PMC5738139; doi:10.1371/journal.pone.0190174)
Supplement: S3 Table — (DOCX) [file pone.0190174.s005.docx]

**S3 Table.** Estimated coefficients (β) and standard errors (SE) for linear and negative binomial regression models comparing species richness and abundance by life-history trait (body size, wing-length, and breeding season) across (log_10_) island area.

|  | Species richness models | | | | | | Abundance models | | | | | |
| --- | --- | --- | --- | --- | --- | --- | --- | --- | --- | --- | --- | --- |
| Variable | Body size | | Wing length | | Breeding season | | Body size | | Wing length | | Breeding season | |
| (code) | β | β SE | β | β SE | β | β SE | β | β SE | β | β SE | β | β SE |
| intercept | 3.765 | 5.019 | 8.933 | 4.968 | 11.146 | 4.732* | -1.799 | 2.424 | -2.100 | 2.457 | 4.957* | 2.280 |
| area | 0.387 | 0.328 | 0.559* | 0.274 | 0.122 | 0.261 | 0.435** | 0.159 | 0.426** | 0.161 | -0.093 | 0.124 |
| canopy cover | 0.007 | 0.011 | -0.102† | 0.056 | -0.080 | 0.053 | 0.012* | 0.005 | 0.014** | 0.005 | -0.035 | 0.025 |
| canopy cover^2 |  |  | 0.001* | <0.001 | 0.001† | <0.001 |  |  |  |  | <0.001† | <0.001 |
| body (small) | 8.818*** | 0.614 |  |  |  |  | 2.486*** | 0.299 |  |  |  |  |
| wing (macro) |  |  | 5.091*** | 0.513 |  |  |  |  | 1.952*** | 0.303 |  |  |
| breeding (spring) |  |  |  |  | 2.669*** | 0.489 |  |  |  |  | 0.559* | 0.233 |
| area x body (small) | -1.522*** | 0.453 |  |  |  |  | -0.704*** | 0.211 |  |  |  |  |
| area x wing (macro) |  |  | -1.729*** | 0.363 |  |  |  |  | -0.800*** | 0.214 |  |  |
| area x breeding (spring) |  |  |  |  | -1.072** | 0.346 |  |  |  |  | -0.236 | 0.165 |
| distance to mainland | 0.165* | 0.068 | 0.151* | 0.058 | 0.146* | 0.055 |  |  |  |  |  |  |
| trap days | -0.006 | 0.009 | -0.010 | 0.008 | -0.009 | 0.007 | 0.008† | 0.004 | 0.008† | 0.004 | 0.001 | 0.003 |
| †p < 0.1, *p < 0.05, **p < 0.01, ***p < 0.001 | |  |  |  |  |  |  |  |  |  |  |  |
