# Supplementary material for: Species traits modify the species-area relationship in ground-beetle (Coleoptera: Carabidae) assemblages on islands in a boreal lake
Source: PLoS One. 2017 Dec 20;12(12):e0190174. doi: 10.1371/journal.pone.0190174 (PMC5738139; doi:10.1371/journal.pone.0190174)
Supplement: S1 Text — (DOCX) [file pone.0190174.s006.docx]

**S1 Text. Carabid identification and voucher locations.**

Adult carabids were identified to species using Lindroth [1] and Bousquet [2]. We included *Trachypachus holmbergi* Mannerheim (Trachypachidae) in the analysis because of its abundance in our study and its similarity and apparently close relationship to carabids [1,3]. Voucher specimens are deposited in the Strickland Museum, Edmonton, Alberta, Canada, and with the collection of the Water Security Agency in Saskatoon, Saskatchewan, Canada.

**References**

**1.** Lindroth CH. The ground-beetles of Canada and Alaska. Opuscula. Entomologica Supplement. 1969; 20, 24, 26, 29, 33, 34, 35: 1-1192.

**2.** Bousquet Y. Illustrated identification guide to adults and larvae of northeastern North American ground beetles *(Coleoptera: Carabidae)*. Pensoft Publishers, Sofia, Bulgaria. Pensoft Series Faunistica No. 90; 2010.

**3.** Bell RT. What is *Trachypachus*? (Coleoptera: Trachypachidae). Coleopt. Bull. 1980; 14: 590-596.
